# Magnesium sulfate for severe preeclampsia and eclampsia management at the primary healthcare level in sub-Saharan Africa: a systematic review

**DOI:** 10.3389/frph.2026.1911083

**Published:** 2026-09-10

**Authors:** Assitan Baya Sidibe, Alexandre Delamou, Fatoumata Bintou Traore, Youssouf Keita, Ben Moulaye Idriss, Aminata Cissé

**Affiliations:** 1 National Office for Reproductive Health (ONASR), Bamako, Mali; 2 African Center of Excellence for Prevention and Control of Communicable Diseases, Conakry, Guinea; 3 National Institute of Public Health (INSP), Bamako, Mali; 4 Global Financing Facility, World Bank Bamako, Bamako, Mali

**Keywords:** eclampsia, hypertensive disorders of pregnancy, magnesium sulfate, maternal mortality, preeclampsia, primary healthcare, sub-Saharan Africa

## Abstract

**Background:**

Preeclampsia and eclampsia are major causes of maternal and perinatal morbidity and mortality in low- and middle-income countries. Magnesium sulfate (MgSO₄) is the most effective intervention for eclampsia prevention and treatment. However, evidence for its use at the primary healthcare level remains limited as most studies have focused on hospital settings.

**Methods:**

This systematic review was conducted in accordance with Preferred Reporting Items for Systematic Reviews and Meta-Analyses guidelines. Electronic databases were comprehensively searched for relevant studies published between January 2003 and December 2024. MEDLINE via PubMed, Cochrane Library, African Index Medicus, and Google Scholar were comprehensively searched for relevant studies. Studies reporting the use of MgSO₄ in primary healthcare settings were included. Data were extracted using a standardized data collection form.

**Results:**

Seven studies, most of which were conducted in Nigeria, met the inclusion criteria. MgSO₄ was available in most primary healthcare facilities but remained underutilized. Task sharing with community health workers was identified as a promising strategy for improving its use. Also, shortcomings were observed in the referral and evacuation system as well as in patient monitoring mechanisms. All studies highlighted the need to strengthen the continuous and regular training and supervision of CHWs to improve early detection and appropriate management of preeclampsia cases.

**Conclusion:**

MgSO₄ administration at the primary healthcare level is a critical intervention for reducing maternal and perinatal mortality associated with preeclampsia and eclampsia. Strengthening task-sharing approaches and community-level integration may enhance health system resilience in sub-Saharan Africa.

## 1 Introduction

Between 1990 and 2021, the global burden of maternal disorders declined substantially; however, marked regional disparities persist, particularly in sub-Saharan Africa. Hypertensive disorders of pregnancy, especially preeclampsia, remain a leading cause of maternal mortality and are associated with a significantly higher age-standardized incidence rate (ASIR) in sub-Saharan Africa than in other regions of the world (1).

Preeclampsia and eclampsia are major contributors to maternal and perinatal morbidity and mortality in low- and middle-income countries (LMICs). The exact prevalence remains uncertain, with substantial variations reported across countries (2). Approximately one-quarter of the stillbirths and neonatal deaths in developing countries are associated with preeclampsia and eclampsia (3).

Most deaths related to preeclampsia in LMICs occur at the community level, highlighting the need for interventions that extend beyond hospital settings and effectively target primary healthcare (4). However, the management of preeclampsia and eclampsia remains challenging in these settings because of the limited availability of essential supplies, shortage of trained healthcare personnel, insufficient competencies among frontline providers, and systemic barriers that delay timely access to appropriate care (5).

Magnesium sulfate (MgSO₄) is widely recognized as the most effective intervention for the prevention and treatment of eclampsia. It is recommended for use in cases of severe preeclampsia and eclampsia, particularly when signs of severity are present (6, 7). The primary objective of MgSO₄ administration is to prevent the onset of eclamptic seizures and reduce associated complications. Additional benefits include reduced maternal and perinatal morbidity and mortality, even among women who do not develop seizures. Furthermore, in cases of mild preeclampsia, MgSO₄ may contribute to reducing progression to severe disease (8).

Despite being listed as an essential medicine, the availability of MgSO₄ varies across healthcare levels, with higher availability in hospitals than in primary healthcare facilities (4, 9). Although coverage for the prevention and treatment of eclampsia has improved, this progress has not consistently translated into adherence to recommended clinical practices across different levels of the health system (10).

Despite its proven effectiveness, low cost, and inclusion on essential medicines lists in most countries, MgSO₄ remains underutilized in many LMICs due to multifaceted barriers (4). Studies conducted in Zambia (11) and the Democratic Republic of the Congo (12) have reported a low uptake of MgSO₄ prophylaxis, poor adherence to clinical guidelines, and significant gaps between recommendations and actual practice in both hospital and primary care settings. Although most health facilities have formal protocols for the management of preeclampsia and eclampsia, their implementation varies geographically with a higher prevalence reported in Latin America than in Africa and Asia (10).

Evidence from the miniPIERS (Pre-eclampsia Integrated Estimate of Risk) model suggests that the use of MgSO₄ in resource-limited settings can significantly improve outcomes, even in contexts where monitoring is minimal or absent (13). In addition, increasing attention has been paid to task-sharing approaches, particularly the role of community health workers (CHWs) in the management of preeclampsia. The World Health Organization recommends optimizing the roles of various healthcare providers, including nurses, midwives, and associate clinicians, in delivering antihypertensive treatment and administering MgSO₄ when appropriate (14).

Existing literature indicates that most studies on MgSO₄ use are conducted at secondary (32.2%) and tertiary (56.2%) healthcare levels, with only a small proportion (11.6%) focusing on primary healthcare and community-based settings (10). However, recent initiatives exploring primary healthcare models in LMICs, such as studies conducted in Bangladesh, Nigeria, and Pakistan, and in-depth assessments in Ethiopia and Kenya, have provided broader insights into access to and delivery of care for preeclampsia and eclampsia (15).

Therefore, this study aims to conduct an exploratory analysis in the sub-Saharan African context to synthesize available evidence on MgSO₄ administration for the management of preeclampsia and eclampsia at the primary healthcare level.

## 2 Methods

### 2.1 Search strategy

This systematic review was conducted in accordance with the Preferred Reporting Items for Systematic Reviews and Meta-Analyses (PRISMA) guidelines (16). The review protocol was developed *a priori* and registered in the International Prospective Register of Systematic Reviews (PROSPERO).

MEDLINE via PubMed, Cochrane Library, African Index Medicus, and Google Scholar were comprehensively searched for relevant studies published from January 1, 2003 to December 31, 2024.

The search strategy combined relevant keywords and controlled vocabulary terms, including Medical Subject Headings (MeSH), to ensure sensitivity and comprehensiveness. Keywords were identified from the titles and abstracts of relevant articles and combined using Boolean operators. Specifically, terms referring to the same concept were combined using “OR,” whereas different concepts were combined using “AND.”

The search strategy was adapted to the syntax and indexing system of each database. Prior to conducting the review, we verified that no similar systematic reviews addressing the same research question had been published by consulting the selected databases and the PROSPERO registry.

The full search strategy for each database is provided in Table 1.

**Table 1:** Search strategy components.

Component	Characteristics
Population	Pregnant women, parturients, and postpartum women
Intervention	Prevention, treatment, and magnesium sulfate (MgSO₄)
Comparator/setting	Primary healthcare level, community health centers, health centers, and hospitals
Outcomes	Severe preeclampsia, eclampsia, maternal death, stillbirth, and neonatal death
Context	Sub-Saharan Africa
Study designs	Quantitative studies, qualitative studies, and randomized controlled trials
Time period	2003–2024
Language	English and French publications

### 2.2 Study selection

A total of 889 records were imported into the Zotero reference management software, and duplicates were removed. Two reviewers independently screened the titles and abstracts based on the predefined inclusion criteria.

Full-text articles that met the inclusion criteria or when there was uncertainty regarding eligibility based on title and abstract screening were retrieved. The eligibility assessment focused on studies addressing the administration of MgSO₄ for severe preeclampsia and eclampsia in sub-Saharan Africa.

Any disagreements between the reviewers were resolved through discussion until a consensus was reached. When necessary, a third reviewer was consulted to adjust for any unresolved discrepancies.

### 2.3 Data extraction

A standardized data extraction form was developed using Microsoft Excel to systematically collect data from the included studies (Table 2). The extracted variables included the country of origin, year of publication, study design, study population, key findings, and study limitations.

**Table 2:** Characteristics of included studies.

Study (Author, Year)	Country	Design	Population	Intervention/Focus	Main findings
Adepoju et al., 2020	Nigeria	Observational	260 patients	MgSO₄ administered by trained CHWs	MgSO₄ administration by trained CHWs was feasible and safe; 95% of patients had no severe complications.
Boene et al., 2019	Mozambique	Cross-sectional	150 CHWs	Knowledge of preeclampsia	Sixty percent recognized preeclampsia signs, but only 25% correctly identified the MgSO₄ dosage.
Oguntunde et al., 2015	Nigeria	Mixed methods	80 providers, 80 facilities, 30 managers	MgSO₄ use	MgSO₄ was recognized as first-line treatment, but its use was limited by inadequate training, stock-outs, lack of protocols, and poor supervision.
Rawlins et al., 2018	Six SSA countries	Multicountry cross-sectional	>4,800 observations; >1,000 providers	Screening and MgSO₄ use	MgSO₄ was available in 60% of facilities and administered in 43% of cases; diazepam was still used in some facilities.
Akeju et al., 2016	Nigeria	Qualitative	30–50 health workers	Task-sharing	Human resource shortages, workload, inadequate training, and limited supervision were the main barriers to task-sharing.
Ishaku et al., 2013	Nigeria	Mixed methods	100–150 records; 20–30 providers	MgSO₄ in primary care	MgSO₄ was feasible for initial stabilization, but improved referral systems and follow-up were required.
Sotunsa et al.	Nigeria	Cross-sectional	∼200 CHWs	CHW knowledge	Additional training and integration of preeclampsia screening into CHW curricula were recommended.

In addition, data extraction focused on identifying gaps related to healthcare provider training in MgSO₄ management, regional disparities in access to MgSO₄, supply and distribution systems in sub-Saharan Africa, and logistical and organizational barriers to its use in primary healthcare settings.

Key variables of interest included provider training, accessibility and availability of MgSO₄, and structural practices related to its implementation in primary healthcare.

Pilot extraction was conducted on a subset of studies to ensure consistency between the reviewers.

### 2.4 Quality assessment of studies

The methodological quality of the studies included was independently assessed by two reviewers using the Critical Appraisal Skills Programme (CASP) checklist (Critical Appraisal Checklists). The following criteria were applied: clarity of study objectives, appropriateness of methodology, relevance of the recruitment strategy, rigor of data collection, adequacy of data analysis, clarity of results, internal and external validity, ethical considerations, practical applicability, and key strengths and limitations (Table 3).

**Table 3:** Quality assessment (CASP summary).

Study	Objective clearly stated	Study design	Recruitment	Data collection	Data analysis	Results clearly reported	Internal validity	External validity	Ethical approval	Applicability	Main strengths	Main limitations
Adepoju et al., 2021	Yes	Observational	Yes	Rigorous	Descriptive	Yes	Good	Moderate	Yes	High	Real-world implementation; demonstrated CHW safety	No control group
Akeju et al., 2016	Yes	Qualitative	Yes	Rigorous	Thematic	Yes	Moderate	Moderate	Yes	Moderate	Comprehensive stakeholder perspectives	Social desirability bias
Boene et al., 2016	Yes	Mixed-methods	Yes	Rigorous	Thematic and statistical	Yes	Moderate	Moderate	Yes	Moderate	Multiple data sources and participant groups	Self-report bias
Ishaku et al., 2013	Yes	Quasi-experimental	Yes	Prospective follow-up	Descriptive and regression	Yes	Moderate	Low–Moderate	Yes	High	Demonstrated feasibility and safety of MgSO₄ use	High loss to follow-up
Oguntunde et al., 2015	Yes	Mixed-methods	Yes	Rigorous	Bivariate and qualitative	Yes	Good	Good	Yes	High	Comprehensive assessment of health-system barriers	Stock-outs not fully quantified
Rawlins et al., 2018	Yes	Cross-sectional	Yes	Direct observation	Descriptive	Yes	Good	Good	Yes	High	Large multicountry assessment	Between-country variability
Sotunsa et al., 2016	Yes	Qualitative	Yes	FGDs and IDIs	Thematic	Yes	Moderate	Moderate	Yes	High	Rich qualitative evidence supporting CHW task-sharing	Context-specific findings

Each study was scored on a 10-point scale and subsequently categorized into three quality levels: low quality (score ≤ 6), moderate quality (score = 7–8), and high quality (score ≥ 9). Any discrepancies between the reviewers were resolved through discussion until a consensus was reached.

Overall, the included studies demonstrated **moderate but acceptable methodological quality**, with important strengths in terms of contextual relevance and diversity of approaches used. However, the overall level of evidence remains **moderate**, mainly due to recurrent limitations such as the lack of rigorous experimental control, selection bias, and methodological heterogeneity.

## 3 Results

**Figure 1:**
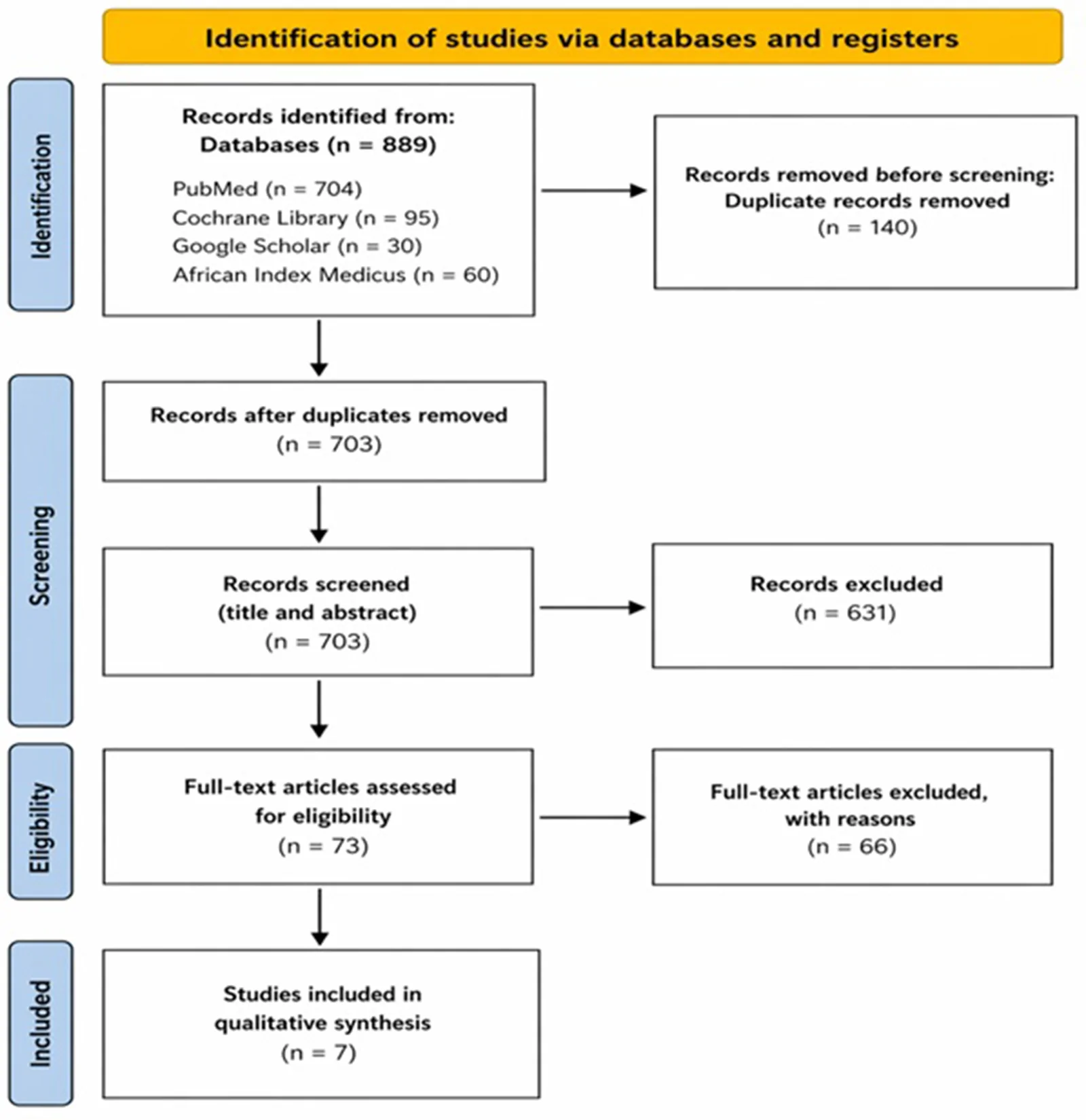
PRISMA 2020 flow diagram of study selecton process.

The database search yielded 889 articles. After removing the duplicates, 703 articles were screened based on their titles and abstracts. Subsequently, 73 full-text articles were assessed for eligibility. Of these, seven met the inclusion criteria and were included in the final systematic review (Figure 1). Sixty-six studies were excluded because they did not focus on the management of severe preeclampsia or eclampsia. Additionally, these studies did not evaluate magnesium sulfate (MgSO₄) use, availability, feasibility, or implementation barriers. Studies conducted exclusively in tertiary healthcare settings without relevance to primary healthcare delivery, as well as those lacking specific data on MgSO₄ management practices.

### 3.1 General characteristics of included studies

Seven studies were included in this systematic review. These studies were conducted in several countries in sub-Saharan Africa: five studies in Nigeria (17–21), one in Mozambique (22), and one multi-country study covering six African countries (Ethiopia, Madagascar, Rwanda, Tanzania, Uganda, and Zambia) (23). The studies were conducted between 2014 and 2022 and various methodological approaches were used. Two were observational studies (17, 23), two were cross-sectional studies (21, 22), two were mixed-method studies (18, 20). These studies included community-based interventions involving CHWs. This heterogeneity reflects both the complexity of implementing MgSO₄-based interventions at the primary healthcare level and the limited number of rigorous experimental studies available in these settings. The study population primarily consisted of CHWs, midwives, and nurses.

### 3.2 Synthesis of results according to main themes

#### 3.2.1 Health system constraints and workforce shortages

In most regions, few healthcare professionals are available at the primary healthcare level, particularly in rural communities. Across all the included studies, a consistent pattern emerged: a critical shortage of qualified health professionals at the primary care level, especially in rural settings. This deficiency may be a determining factor in the management of preeclampsia and eclampsia. Consequently, CHWs perform some assigned tasks. Thus, in many contexts, CHWs effectively play the role of frontline providers, often compensating for the absence or limited availability of nurses and midwives.

Task shifting may be a pragmatic solution to address these limitations, by assigning certain responsibilities to CHWs (screening, initial MgSO₄ administration, and referral). This strategy improved the coverage of essential interventions and reduced maternal mortality in several similar contexts. However, the effectiveness of this approach depends on its integration into a broader health system framework. Without structured supervision, continuous training, or functional referral pathways, task shifting alone is unlikely to produce sustainable improvements in outcomes.

#### 3.2.2 Knowledge and training of health providers

The data revealed significant variability in the knowledge and skills of the frontline healthcare providers. In Ogun State (Nigeria), CHWs had low overall knowledge levels; fewer than half were able to correctly identify danger signs such as hypertension, severe headaches, or proteinuria, and only 38.3% had been trained in preeclampsia and eclampsia in health facilities, whereas 90% had not received supervisory support (Sotunsa et al.). Another study has reported that 57.8% of CHWs were trained in Ogun State (Adepoju et al., 2021).

Similarly, in Mozambique, CHWs were aware of the condition but lacked in-depth clinical training to ensure proper detection and referral. Although 60% reported receiving regular training to identify early pregnancy complications, very few (5%) felt confident in administering injections of any kind (Boene et al., 2016).

This gap is context-dependent, reflecting differences in training systems, supervision structures, and national policies. Thus, training coverage is uneven and even when it exists, it does not always translate into confidence or practical competence, particularly for clinical tasks such as injection administration.

All studies highlighted the need to strengthen the continuous and regular training and supervision of CHWs to improve early detection and appropriate management of preeclampsia cases.

#### 3.2.3 Availability and use of MgSO₄

To improve the availability and accessibility of MgSO₄ in low-income countries, several strategies have been developed, considering the specific context of each setting and WHO recommendations.

An Ogun State study involving 260 treatments initiated by CHWs showed that they could safely administer MgSO₄ under minimal supervision without major adverse events (Adepoju et al., 2021). In the multicountry survey, MgSO₄ was administered in only 43% of cases, and all treated women were successfully managed, with no reported cases of MgSO₄ toxicity (Rawlins et al.). The study titled “Abandonment of care after the critical phase” highlighted post-critical loss to follow-up, with some women leaving care after stabilization, underscoring the need for strengthened referral and follow-up systems (20).

The main finding across these studies is that MgSO₄ administration at primary and community health care levels is feasible and can be safely performed by trained CHWs under appropriate supervision. However, its feasibility depends strongly on the presence of a minimum level of health support, including training, supervision, and clear clinical protocols.

Regarding availability, a study from northern Nigeria has indicated that MgSO₄ was available in most health facilities (63.4%) (18). In a multicountry survey, availability in health centers and clinics was up to 70% (23).

A recurring finding is the mismatch between the availability of MgSO_4_ and its clinical use. Although this drug is available in a substantial proportion of healthcare facilities, it remains underutilized. Thus, improving the supply alone may be insufficient. The continued use of less effective alternative treatments such as diazepam in some contexts further illustrates this implementation gap.

#### 3.2.4 Structural and organizational practices

The included studies highlighted multiple interacting barriers limiting the effective use of MgSO_4_ at the primary healthcare level. These barriers include irregular drug supply chains and stockouts, a lack of standardized treatment protocols, insufficient supervision and mentorship, and limited system integration. This indicates that the challenge is not only clinical, but also deeply rooted in health system organization and governance.

However, in settings where full MgSO₄ treatment regimens cannot be administered, World Health Organization guidelines recommend giving women with severe preeclampsia or eclampsia an initial loading dose of MgSO₄ and then immediately transferring them to a higher-level health facility, with care provided by midwives and nurses (24).

A multicountry study conducted in six sub-Saharan African countries revealed highly variable management of preeclampsia and eclampsia in antenatal services. In most facilities observed, protocols were not systematically followed, MgSO₄ was underused, and blood pressure monitoring was often irregular. The findings also showed that a lack of material resources and continuous training limited the quality of care, particularly in rural facilities (23).

#### 3.2.5 Continuity of care

Gaps in continuity of care, particularly after initial stabilization with MgSO₄, and loss to follow-up observed after treatment highlight weaknesses in referral and evacuation systems as well as patient tracking mechanisms (17, 20, 25, 26). Although MgSO₄ administration at primary care level may address immediate clinical needs, long-term outcomes depend on effective coordination between primary health facilities and referral hospitals.

## 4 Discussion

This systematic review synthesizes evidence from seven studies conducted in sub-Saharan Africa, primarily in Nigeria and Mozambique, and demonstrates that MgSO₄ administration for the management of severe preeclampsia and eclampsia at the primary and community levels is both feasible and safe when implemented under appropriate conditions. However, beyond clinical effectiveness, the findings consistently highlight that the major bottleneck lies in health system capacity to ensure consistent and appropriate use of MgSO₄.

A central finding of this review is the persistent gap between MgSO₄ availability and its actual use in clinical practice. Despite being listed as an essential medicine in many primary healthcare facilities, its utilization remains suboptimal. This gap in implementation suggests that improving drug availability alone is insufficient. Instead, systemic barriers, including inadequate training, low provider confidence, weak adherence to clinical protocols, and insufficient supervision—play a decisive role in limiting effective use.

Across the included studies, task sharing with CHWs emerged as a promising strategy for improving early detection and timely management. Their involvement is consistently associated with improved identification of danger signs and reduced delays in initiating treatment. However, this potential is constrained by the substantial variability in training, competencies, and roles across countries. For example, Nigerian CHWs include nurses, midwives, and community-based cadres (17), whereas Mozambique relies on formally designated community agents (APEs) with heterogeneous educational backgrounds (22). These contextual differences underscore the importance of adapting task-sharing strategies to local health-system structures.

Importantly, the review revealed that many frontline providers, particularly at the community level, have a limited ability to recognize the key clinical signs of preeclampsia. In several settings, fewer than half of the providers correctly identified major symptoms, such as hypertension, proteinuria, or severe headaches. Similar gaps have been reported in other low- and middle-income countries, including Pakistan, India, and Bangladesh (27, 28), suggesting that this is a broader systemic issue than a context-specific problem. These deficiencies highlight the urgent need for standardized competency-based training programs combined with ongoing supervision and mentorship.

Regarding MgSO₄ administration, the evidence indicates that trained CHWs can safely initiate treatment under minimal supervision (17, 20, 26). This finding is particularly important for improving access to lifesaving care in underserved areas. However, this contrasts with findings from some settings where awareness and use of MgSO₄ remain limited among lower-level providers, and concerns regarding safety persist (28, 29). This discrepancy highlights the importance of not only training but also addressing perceptions, regulatory barriers, and the scope of practice limitations.

The review further shows that underutilization of MgSO₄ cannot be explained solely by resource constraints. Although stockouts and supply chain weaknesses remain relevant, deeper structural issues are evident, including fragmented governance, lack of standardized treatment protocols, weak referral systems, and inadequate integration between levels of care (23, 30). Despite clear clinical guidelines, the continued use of less effective alternatives, such as diazepam, illustrates persistent gaps in guideline dissemination and implementation.

Continuity of care has emerged as a critical weakness. Although primary-level administration of MgSO₄ can stabilize patients, the benefits are often undermined by failures in referral and follow-up systems. Loss to follow-up after initial stabilization reflects broader deficiencies in health system coordination, suggesting that improving outcomes requires strengthening the entire continuum of care, rather than focusing solely on initial treatment.

The included studies show that primary healthcare facilities in sub-Saharan Africa operate under significant resource constraints that hinder the effective management of severe preeclampsia and eclampsia. Frequent shortages of magnesium sulfate, essential diagnostic equipment, trained personnel, and standardized clinical protocols, together with weak referral systems and inadequate supportive supervision, limit the capacity of frontline healthcare providers to deliver timely, evidence-based care Nevertheless, the studies consistently demonstrate that with appropriate training, supportive supervision, and a reliable supply of magnesium sulfate, frontline providers can safely administer the loading dose before referral, highlighting that health system strengthening is essential for improving maternal outcomes.

Although this review provides important insights, its findings must be interpreted considering the limitations of the included studies. The available evidence was mainly derived from observational and qualitative studies, with a limited number of countries represented, which may reduce the generalizability of findings. Studies reporting successful implementation experiences may have been more likely to be published, while unsuccessful practices or implementation barriers may have been underreported. Therefore, the findings should be interpreted with caution.

The heterogeneity among the seven included studies should be considered when interpreting the findings. Differences in study designs (cross-sectional, mixed-methods, and qualitative approaches), study populations, healthcare settings, and health system contexts may have influenced the reported availability, use, and implementation of magnesium sulfate at the primary healthcare level. Variations in intervention strategies, including differences in training approaches, provider categories involved, supervision mechanisms, and referral practices, also contributed to differences in outcomes across studies. In addition, the diversity of outcome measures, ranging from knowledge and provider practices to drug availability and maternal care processes, limited direct comparisons between studies. Despite this variability, the findings consistently highlight common challenges related to health system capacity, provider preparedness, and access to essential medicines.

Although this review provides important insights, its findings must be interpreted considering the limitations of the included studies. The predominance of observational and qualitative designs, reliance on self-reported data, and heterogeneity across contexts limit the causal inferences. Nevertheless, the consistency of the findings across diverse settings strengthens confidence in the overall conclusions.

Overall, this review underscores the need to move beyond a purely biomedical perspective toward a system-based approach to improve maternal outcomes related to preeclampsia and eclampsia in sub-Saharan Africa. Strengthening primary healthcare systems, institutionalizing task-sharing policies, ensuring continuous training and supervision, and improving referral and supply chain systems are essential for maximizing the impact of MgSO_4_. Future research should prioritize the implementation of scientific approaches and context-specific evaluations to identify scalable and sustainable models for integrating MgSO₄ into routine primary care practice.

### 4.1 Strengths and limitations

#### 4.1.1 Strengths

This systematic review provides a comprehensive synthesis of the available evidence on the use of MgSO₄ for the management of preeclampsia and eclampsia at the primary healthcare level in sub-Saharan Africa. A key strength of this study lies in its focus on community and primary care settings, which remain underrepresented in literature despite their critical role in maternal health outcomes.

The review was conducted using a rigorous methodological approach in accordance with the PRISMA guidelines, including a structured search strategy across multiple databases and independent screening and data extraction by reviewers. The inclusion of diverse study designs and quantitative, qualitative, and mixed methods allowed for a nuanced and comprehensive understanding of the clinical practices and health system challenges.

Furthermore, the use of the CASP framework for quality assessment enhanced methodological transparency and reliability of the findings. The consistency of the results across different countries and study designs strengthens the credibility of the evidence and supports the external validity of the conclusions.

#### 4.1.2 Limitations

This study has some limitations. First, the predominance of observational, cross-sectional, and qualitative studies limits our ability to establish a causal relationship between MgSO4 use and maternal and perinatal outcomes. The absence of large-scale randomized controlled trials further reduced the overall strength of the evidence.

Second, heterogeneity in the study design, population, and outcome measures limits comparability across studies and precludes the use of a quantitative meta-analysis. Consequently, the synthesis relied on a narrative approach, which may be subject to interpretative bias.

Third, In the literature reviewed, most studies were conducted in reference health centers or hospitals, which led to the selection of 7 studies. Most included studies were conducted in specific geographical settings, particularly in Nigeria, which may limit the generalizability of the findings to other sub-Saharan African contexts with different health system structures.

Additionally, the reliance on self-reported data from healthcare providers introduces the risk of reporting and social desirability bias. Finally, inconsistencies in data collection methods and the absence of control groups in some studies may have affected the internal validity of the findings.

### 4.2 Future implications

The use of MgSO₄ in the management of severe preeclampsia and eclampsia at the primary health care level in sub-Saharan Africa is aligned with the achievement of the Sustainable Development Goals.

The findings of this review have important implications for health policy and the organization of maternal health services in sub-Saharan Africa. Although MgSO₄ is widely recognized as an essential and effective intervention for the management of preeclampsia and eclampsia, this review highlights that the main challenges lie primarily in its implementation within primary health care systems. Therefore, national policies that explicitly support the decentralization of preeclampsia and eclampsia management at primary health care levels need to be developed. Integrating MgSO₄ administration into routine primary care services, particularly in rural and underserved areas, could significantly reduce treatment delays and improve maternal outcomes.

In addition, task-shifting policies should be strengthened and formalized by expanding the scope of practice of CHWs to include the initial management of severe preeclampsia, supported by clear regulatory frameworks, standardized clinical guidelines, and certification systems.

Investing in healthcare workforce capacity building is also necessary. This includes not only initial training, but also continuous professional development, high-quality supervision, and mentoring systems for frontline providers. Without sustained capacity-building efforts, the effectiveness of task shifting strategies is likely to remain limited.

Further, supply chain systems should be strengthened to ensure the continuous availability of MgSO₄ at all levels of care. Policies should prioritize procurement planning, distribution systems, and monitoring mechanisms to guarantee uninterrupted access.

Referral and evacuation systems and continuity of care are also necessary. Policymakers should strengthen the coordination among community structures, primary health facilities, and referral hospitals.

Moreover, monitoring and evaluation systems should be reinforced by integrating relevant indicators into national health information systems to better assess the coverage, quality of care, and health outcomes.

Overall, the large-scale implementation of MgSO₄ use at the primary healthcare level requires a systemic approach that combines policy reform, workforce development, supply chain strengthening, and community engagement. These integrated strategies are essential for accelerating progress in reducing maternal and perinatal mortality in sub-Saharan Africa.

## 5 Conclusions

Practices related to MgSO₄ use for the prevention and treatment of eclampsia at the primary healthcare level vary considerably across countries. This review highlights significant challenges faced by these countries, including limited use of MgSO₄ at the primary care level, persistent constraints in training and supervision, and poor adherence to clinical protocols by most health care providers.

Task shifting and community-based integration into primary healthcare have emerged as sustainable strategies to strengthen the resilience of health systems in Sub-Saharan Africa.

This review provides a knowledge base that can contribute to the development of effective primary healthcare strategies for the use of MgSO₄ in sub-Saharan African countries. Its findings may guide interventions in key areas such as improving MgSO₄ use among frontline providers, particularly CHWs, strengthening health workers' knowledge, ensuring continuous supervision, and expanding task delegation. Addressing these interrelated challenges is essential to reduce maternal and perinatal mortality associated with preeclampsia and eclampsia, and to advance global maternal health goals in sub-Saharan Africa.
